# Magnetotactic Coccus Strain SHHC-1 Affiliated to *Alphaproteobacteria* Forms Octahedral Magnetite Magnetosomes

**DOI:** 10.3389/fmicb.2017.00969

**Published:** 2017-05-30

**Authors:** Heng Zhang, Nicolas Menguy, Fuxian Wang, Karim Benzerara, Eric Leroy, Peiyu Liu, Wenqi Liu, Chunli Wang, Yongxin Pan, Zhibao Chen, Jinhua Li

**Affiliations:** ^1^Department of Life Science and Technology, Heilongjiang Bayi Agricultural UniversityDaqing, China; ^2^Key Laboratory of Earth and Planetary Physics, Institute of Geology and Geophysics, Chinese Academy of SciencesBeijing, China; ^3^Laboratory for Marine Geology, Qingdao National Laboratory for Marine Science and TechnologyQingdao, China; ^4^France-China Biomineralization and Nano-structures Laboratory, Chinese Academy of SciencesBeijing, China; ^5^IMPMC, Centre National de la Recherche Scientifique, UMR 7590, Sorbonne Universités, MNHN, UPMC, IRD UMR 206Paris, France; ^6^France Chimie Me′tallurgique des Terres Rares, ICMPE, UMR 7182, Centre National de la Recherche ScientifiqueThiais, France

**Keywords:** magnetotactic cocci, magnetosome, biomineralization, octahedron, coordinated FISH-SEM, TEM

## Abstract

Magnetotactic bacteria (MTB) are morphologically and phylogenetically diverse prokaryotes. They can form intracellular chain-assembled magnetite (Fe_3_O_4_) or greigite (Fe_3_S_4_) nanocrystals each enveloped by a lipid bilayer membrane called a magnetosome. Magnetotactic cocci have been found to be the most abundant morphotypes of MTB in various aquatic environments. However, knowledge on magnetosome biomineralization within magnetotactic cocci remains elusive due to small number of strains that have been cultured. By using a coordinated fluorescence and scanning electron microscopy method, we discovered a unique magnetotactic coccus strain (tentatively named SHHC-1) in brackish sediments collected from the estuary of Shihe River in Qinhuangdao city, eastern China. It phylogenetically belongs to the *Alphaproteobacteria* class. Transmission electron microscopy analyses reveal that SHHC-1 cells formed many magnetite-type magnetosomes organized as two bundles in each cell. Each bundle contains two parallel chains with smaller magnetosomes generally located at the ends of each chain. Unlike most magnetotactic alphaproteobacteria that generally form magnetosomes with uniform crystal morphologies, SHHC-1 magnetosomes display a more diverse variety of crystal morphology even within a single cell. Most particles have rectangular and rhomboidal projections, whilst others are triangular, or irregular. High resolution transmission electron microscopy observations coupled with morphological modeling indicate an idealized model—elongated octahedral crystals, a form composed of eight {111} faces. Furthermore, twins, multiple twins and stack dislocations are frequently observed in the SHHC-1 magnetosomes. This suggests that biomineralization of strain SHHC-1 magnetosome might be less biologically controlled than other magnetotactic alphaproteobacteria. Alternatively, SHHC-1 is more sensitive to the unfavorable environments under which it lives, or a combination of both factors may have controlled the magnetosome biomineralization process within this unique MTB.

## Introduction

Magnetotactic bacteria (MTB) are a group of geographically widespread prokaryotes sharing the common ability to form magnetite (Fe_3_O_4_) or greigite (Fe_3_S_4_) nanocrystals intracellularly, each surrounded by a lipid bilayer membrane (Bazylinski and Frankel, 2004). These intracellular magnetic particles are called magnetosomes that generally arrange into chain structures (Balkwill et al., 1980). They serve as a cellular biocompass, enabling the MTB cells to passively align along the geomagnetic field lines. Using this compass, MTB can actively swim, to their favorite niches at or just below the oxic-anoxic interface (OAI) in aquatic environments (Frankel et al., 1997; Bazylinski and Frankel, 2004). This process is termed as magneto-aerotaxis (Frankel et al., 1997; Lefèvre et al., 2014). In order to better understand magneto-aerotaxis, the study of the diversity and biomineralization of MTB is required. Moreover, a more comprehensive understanding of MTB is required for the potential application of magnetosomes in biotechnological and biomedical fields (Faivre and Schüler, 2008; Li et al., 2013; Pósfai et al., 2013a). Fossilized remains of MTB (i.e., magnetofossils) have also been tentatively used to retrieve paleomagnetic and paleoenvironmental information from ancient sediments (Hesse, 1994; Yamazaki and Kawahata, 1998; Snowball et al., 2002; Roberts et al., 2011; Larrasoana et al., 2014; Liu et al., 2015), and to trace the origin and evolution of life on Earth and even perhaps Mars (Chang and Kirschvink, 1989; McKay et al., 1996; Thomas-Keprta et al., 2002).

MTB that have been detected so far have many morphotypes, including spirillum, coccus, vibrio, ovoid, rod-shaped, and even multicellular bacteria. These known cultured and uncultured MTB have been assigned to the *Alpha*-, *Gamma*-, and *Delta-proteobacteria* classes of the *Proteobacteria* phylum (Lefèvre and Bazylinski, 2013), the *Nitrospirae* phylum (Spring et al., 1993), and even the “*Candidatus* Omnitrophica” phylum (Kolinko et al., 2012). Despite the morphological and phylogenetic diversity of MTB, the most commonly observed types present in natural environments are coccoid-to-ovoid cells, the so-called magnetococci (Moench and Konetzka, 1978; Spring et al., 1992, 1995; Thornhill et al., 1995; Cox et al., 2002; Flies et al., 2005; Pan et al., 2008, 2009; Lin and Pan, 2009; Zhang et al., 2012; Chen et al., 2015; Abreu et al., 2016). Previous 16S rDNA phylogenetic analyses have shown that magnetococci are also highly diverse. Most magnetococci are not closely related to other *Alphaproteobacteria* and form an independent clade within the *Alphaproteobacteria* (i.e., the *Magnetococcales* order) that is basal to the rest of the group (Bazylinski et al., 2013). This phylogenetic diversity is consistent with a broad morphological diversity of magnetosomes among different species or strains of magnetococci. In particular, the chain assemblages of magnetosomes are diverse, e.g., a single chain (Lefèvre et al., 2009; Pan et al., 2009; Bazylinski et al., 2013), double chains (Pan et al., 2008), multiple chains (Lin et al., 2009), and dispersed aggregates or clusters (Moench and Konetzka, 1978; Flies et al., 2005; Lin and Pan, 2009; Zhang et al., 2012) can occur. Many magnetococci contain sulfur globules and/or calcium polyphosphate inclusions in addition to magnetosomes within a single cell, suggesting their great potential for iron, sulfur, and phosphorus cycling in natural environments (Cox et al., 2002; Araujo et al., 2016). Despite the wide distribution and high diversity of MTB, only three magnetococci strains have been axenically cultured so far: the *Magnetococcus marinus* strain MC-1 (Bazylinski et al., 2013), the MO-1 strain (Lefèvre et al., 2009), and the *Magnetofaba australis* stain IT-1 (Morillo et al., 2014). They all form magnetosomes organized as a single chain. In addition, the spatial arrangements and crystal morphologies of magnetosomes remain poorly documented for many uncultured and yet phylogenetically constrained magnetococci.

In order to expand our knowledge of the diversity of magnetococci and the crystallographic properties of their magnetosomes, we studied a unique magnetotactic coccus tentatively named strain SHHC-1 from brackish sediments collected in the estuary of the Shihe River in Qinhuangdao City, eastern China. In order to characterize it, we used a recently developed method called coupled FISH-SEM (Li et al., 2017). This method enabled the rapid phylogenetic and biomineralogical characterization of uncultured MTB at the single-cell level by coordinating fluorescence with scanning electron microscopy (SEM) (Li et al., 2017). Transmission electron microscopy (TEM) analyses were further performed on this magnetococcus to reveal the crystallographic features of its magnetosomes in high detail.

## Materials and methods

### Sediment sampling and sample preparation

**Figure 1:**
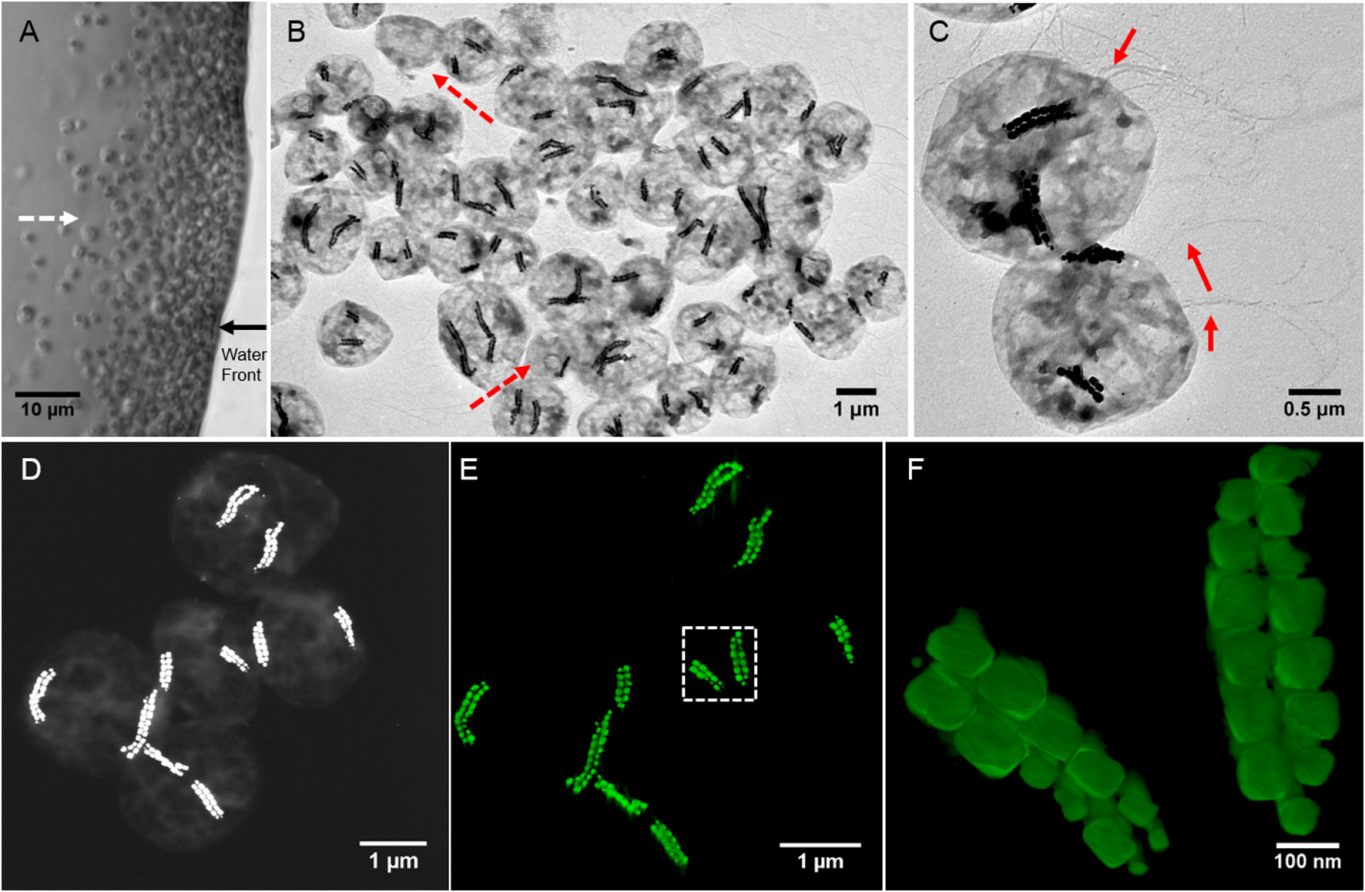
Morphological features of SHHC-1 cells. **(A)** Optical microscopy image of living SHHC-1 cells swimming out from one small drop of sediment on the left (photograph not shown) along the applied magnetic field lines (indicated by the white dashed line arrow), and accumulating at the edge of the water droplets (indicated by the black solid line arrow). **(B)** Low-magnification TEM image of SHHC-1 cells collected from sediments. The red dash line arrows indicate other types of MTB or non-MTB cells. **(C)** TEM image of two SHHC-1 cells showing their magnetosome chains and flagella (indicated by the red line arrows). **(D)** HAADF-STEM image of five SHHC-1 cells for HAADF-STEM tomography observations. **(E)** HAADF-STEM tomographic image (three-dimensional visualization) showing the spatial arrangement of double bundles of magnetosome chains within the five SHHC-1 cells shown in **(D)**. **(F)** HAADF-STEM tomographic image of magnetosome chains within the SHHC-1 cell indicated by the white dashed box in **(E)**.

The MTB-bearing sediment samples were collected from the estuary of the Shihe River in Qinhuangdao city, eastern China. The sampling site was located inside the estuary of Shihe River into the Bohai Sea (39°57′55.6″N, 119°47′9.4″E). The sediment comes from a brackish lacustrine environment with a salinity of ~23.9 ppt, a pH of 7.5 and a temperature of 19°C measured in July 2015. Surface sediments were collected near the shore at water depths of ~1–2 meters and transferred to 1-l plastic bottles with a sediments: water ratio of ~2:1. The bottles were shipped back to the laboratory and stored at room temperature (~20°C) in dim light to set up microcosms. Magnetotacic bacteria in the microcosms were routinely checked with the hanging-drop technique (Schüler, 2002) using an Olympus microscope BX51 equipped with phase-contrast, fluorescence and a DP70 digital camera system (Olympus Corp., Tokyo, Japan). Although they were collected from the same sample site, MTB populations differed among individual microcosms after several weeks of incubation in the laboratory, and even varied within the same microcosm with time (Li et al., 2017). Here, we focused on one microcosm dominated by one group of magnetococci (i.e., SHHC-1) that swam toward the south pole of a bar magnet (north-seeking MTB) (Figure 1A).

Approximately 200 ml of slurry from this microcosm was used to magnetically concentrate the SHHC-1 cells by using a modified magnetic separation apparatus previously described in Jogler et al. (2009). The collected MTB cells were washed three times with Milli-Q water and then re-suspended into ~100 μl of Milli-Q water for additional experiments: ~5 μl of MTB cells were used for TEM analyses, ~20 μl were boiled for 10 min and stored at −20°C for PCR amplification of 16S rRNA genes, and the remaining ~75 μl were prepared for coupled FISH-SEM analyses as previously described in Li et al. (2017). TEM samples were stored in a pure N_2_ atmosphere prior to TEM observations.

### Phylogenetic analyses and bacterial identification

SixteenS rRNA genes of the MTB cells were amplified using the universal bacterial primers 27F (5′-AGAGTTTGATCCTGGCTCAG-3′) and 1492R (5′-GGTTACCTTGTTACGACTT-3′) (Lane, 1991), and sequenced using protocol described in Li et al. (2017). Briefly, each 50 μl PCR mixture contained 1 μl of template, 25 μl of DreamTaq PCR Master Mix (MBI Fermentas), 2 μl of each primer (10 μM), and 20 μl of Milli-Q water. The PCR conditions were 95°C for 3 min, 30 cycles at 95°C for 1 min, 55°C for 1.5 min, 72°C for 1.5 min, and a final 10 min extension at 72°C. To avoid potential amplification biases, triplicate PCR products from each sample were pooled and purified using 0.8% (w/v) agarose gel electrophoresis (described below). All PCR controls without added template were negative. PCR products were purified using an E.Z.N.A.® Gel Extraction Kit (Omega Bio-tek, Inc. USA). They were ligated with the pMD19-T vector (TaKaRa, Japan), then cloned in *Escherichia coli* (strain DH5α) competent cells (Tiangen, Beijing, China) according to the manufacturer instructions. Thirty clones were randomly picked and sequenced using the vector primers M13-47 (5′-CGCCAGGGTTTTCCCAGTCACGAC-3′) and RV-M (5′-GAGCGGATAACAATTTCACACAGG-3′) at the Huada Genome Center (Beijing, China). After discarding sequences of insufficient length (<1,000 bp), the remaining sequences were then aligned with their close relatives using the ClustalW algorithm for manual correction, and a phylogenetic tree was subsequently constructed using the neighbor-joining method (Saitou and Nei, 1987) in the MEGA software package (v. 7.0) (Kumar et al., 2016). Bootstrap values were calculated with 1,000 replicates.

To phylogenetically and morphologically identify the SHHC-1 cells, the probe SHHC1228 (5′-CTCCAGGTCACCCATTCGCCGCTCT-3′, positions 1,228–1,252) was designed to target specifically all 16S rRNA gene sequences identified in this study. Probe specificity was evaluated by using the online probe evaluation tools probeCheck and probeBase (Loy et al., 2008; Greuter et al., 2016). No other sequence in the SILVA111 database has a matching complementary sequence (the minimum number of mismatches is 2). The universal bacterial probe EUB338 (5′-GCTGCCTCCCGTAGGAGT-3′) was used as a positive control probe of bacteria for FISH (Amann et al., 1990). Probe EUB338 was synthesized and fluorescently labeled with fluorescein phosphoramidite FAM at the 5′ end, while the SHHC1228 probe was synthesized and fluorescently labeled with the hydrophilic sulfoindocyanine dye Cy3 at the 5′ end. *E. coli* cells were added in approximately same amount to the SHHC-1 cells and were used as inner control cells of non-magnetotactic bacteria.

Coordinated FISH-SEM analysis was carried out using the procedure described in Li et al. (2017). Briefly, ~1 μl of cell mixtures (*E. coli* + SHHC-1) were placed directly on a high-precision cover glass (Paul Marienfeld GmbH & Co. KG, Germany) and dried in air at ambient temperature, followed by gradual dehydration in 50, 80, and 100% ethanol baths (3 min in each bath). *In situ* hybridization was performed at 46°C for 3 h in 9 μl of hybridization buffer (0.9 M NaCl, 20 mM Tris-HCl [pH 7.5], 0.02% [wt/vol] sodium dodecyl sulfate [SDS], 35% [vol/vol] formamide). One microliter of the EUB338 probe (50 ng/μl) and 1 μl of the SHHC1228 probe of interest (50 ng/μl) was then added to the mixture. After 3 h of hybridization the samples were incubated in a washing buffer (0.08 M NaCl, 20 mM Tris-HCl [pH 7.5], 5 mM EDTA [pH 8.0], 0.01% [wt/vol] SDS) at 48°C for 30 min, then washed in Milli-Q water three times, dried in air at ambient temperature and finally observed using an Olympus epifluorescent microscope BX51. After fluorescence microscopy observations, the cells mounted on cover glasses were coated with carbon using a Leica ACE200 Low Vacuum Sputter Coater (Leica Microsystems, Wetzlar, Germany), and observed using a Zeiss Ultra-55 field-emission gun scanning electron microscope (Carl Zeiss, Germany) operating at 5 kV.

### Transmission electron microscopy analyses

Conventional TEM observations were performed on a JEM2100 microscope (JEOL Ltd., Tokyo, Japan) operating at 200 kV at the IGG-CAS (Beijing, China). High resolution TEM (HRTEM) observations were carried out on a JEM-2100F microscope (JEOL Ltd., Tokyo, Japan) operating at 200 kV at the IMPMC (Paris, France). This microscope was equipped with a field emission gun, an ultra-high resolution (UHR) pole piece, a JEOL detector with an ultrathin window, and a scanning TEM (STEM) device. STEM Z-contrast images were acquired in high angle annular dark field (HAADF) mode. Chemical composition analyses were performed by energy dispersive X-ray spectrometry (EDXS) elemental mapping in HAADF-STEM mode. HAADF-STEM tomography was performed on a FEI Tecnai F20 microscope at 200 kV at the ICMPE (Thiais, France). Acquisition was made using the Digital MicrographTM (Gatan) STEM tomography module, and 3-D reconstruction was performed using the DigiECT software (available at http://www.digisens3d.com/fr/logiciel-tomographie/soft/23D_Electron_Tomography_Software.html).

The crystal length (along the long axis) and width (perpendicular to the long axis) of the magnetosomes were measured from the TEM images. The shape factor was defined as width/length. Crystal habits of magnetosomes were determined by a combination of Fourier analyses of HRTEM images of individual particles and crystallographic investigations, as previously described in Faivre et al. (2008) and Li et al. (2015). For each particle, the zone axis and in plane crystallographic directions were determined from the 2-D Fast Fourier transform (FFT) of the HRTEM image, enabling the the stereographic projection to be determined. Idealized shapes modeled using the KrystalShaper software package (available at http://www.jcrystal.com/) were then compared to the observed ones.

## Results

### Morphology and ultrastructure of SHHC-1 cells

TEM observations showed that the magnetically concentrated cells contained predominantly one group of magnetococci, called SHHC-1, with two bundles of magnetosome chains, and a few (< 5%) other magnetotactic cocci forming single magnetosome chain or non-MTB cells (Figure 1B). The SHHC-1 cells were coccoid in shape with an average diameter of 2.1 ± 0.4 μm (*n* = 150). They contained two bundles of flagella, and with an average magnetosome number of 38 ± 17 per cell (*n* = 150) (Figure 1C). HAADF-STEM tomography observations clearly evidenced that each bundle was composed of two parallel closely assembled magnetosome chains (Figures 1D–F and Supplementary movie). Smaller magnetosomes were often observed at the ends of the chains. Within the same SHHC-1 cell, the two bundles of magnetosome chains were commonly distributed on opposite sides of the cell body in close proximity to the cell membrane. Such an arrangement of the magnetosomes in multiple chains supports the previous assumption that repulsion forces between parallel magnetic dipoles may drive the chains or bundle of chains apart from one another. Thus, the magnetic torque acting on them by the external applied or the geomagnetic field can be effectively transferred to the entire cell body (Hanzlik et al., 1996). In the case of SHHC-1, each bundle of chains may behave as one enlarged SD particle because all the individual magnetosomes may have their magnetic moments uniformly aligned along the chain axis, as previously shown by electron holography observations performed on another uncultured, phylogenetically unknown, magnetococcus strain having two bundles of magnetosome chains (Simpson et al., 2005).

**Figure 2:**
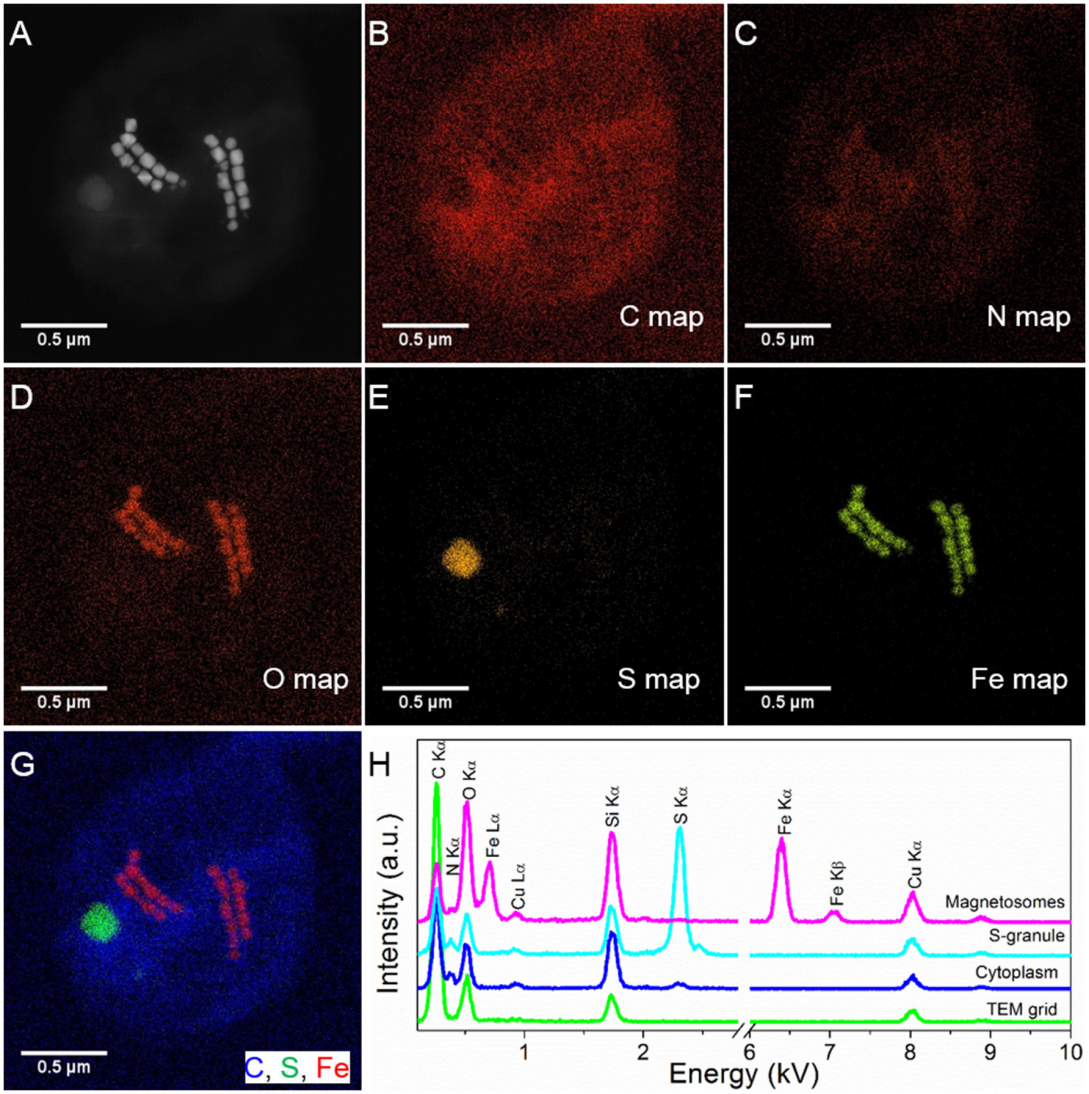
STEM-EDXS mapping (HAADF mode) of SHHC-1 cells. **(A–F)** HAADF-STEM image of a single SHHC-1 cell **(A)**, and the corresponding chemical maps of C (C Kα) **(B)**, N (N Kα) **(C)**, O (O Kα) **(D)**, S (S Kα) **(E)**, and Fe (Fe Kα) **(F)**. **(G)** Overlapped map of Fe (red), C (blue), and S (green). **(H)** EDXS spectra extracted from the carbon film covering TEM grid (green line), cell wall (blue line), sulfur-rich particle (light blue line), and magnetosomes (pink line).

STEM-EDXS mapping (in HAADF mode) clearly shows that the SHHC-1 magnetosomes are rich in Fe and O, consistently with magnetite. Sulfur-rich globules with various sizes, ranging from several to hundreds of nanometers were also often observed within the SHHC-1 cells (Figure 2). These features were consistent with previous observations of many uncultured magnetococci that can form sulfur globules as well as magnetite-type magnetosomes, even when sulfide is absent in the sediment sample (Moench, 1988; Cox et al., 2002; Araujo et al., 2016). This confirmed that most of magnetococci cells may have an autotrophic or mixotrophic metabolism based on the oxidization of reduced sulfur compounds (Araujo et al., 2016).

### Phylogeny of the SHHC-1 strain

**Figure 3:**
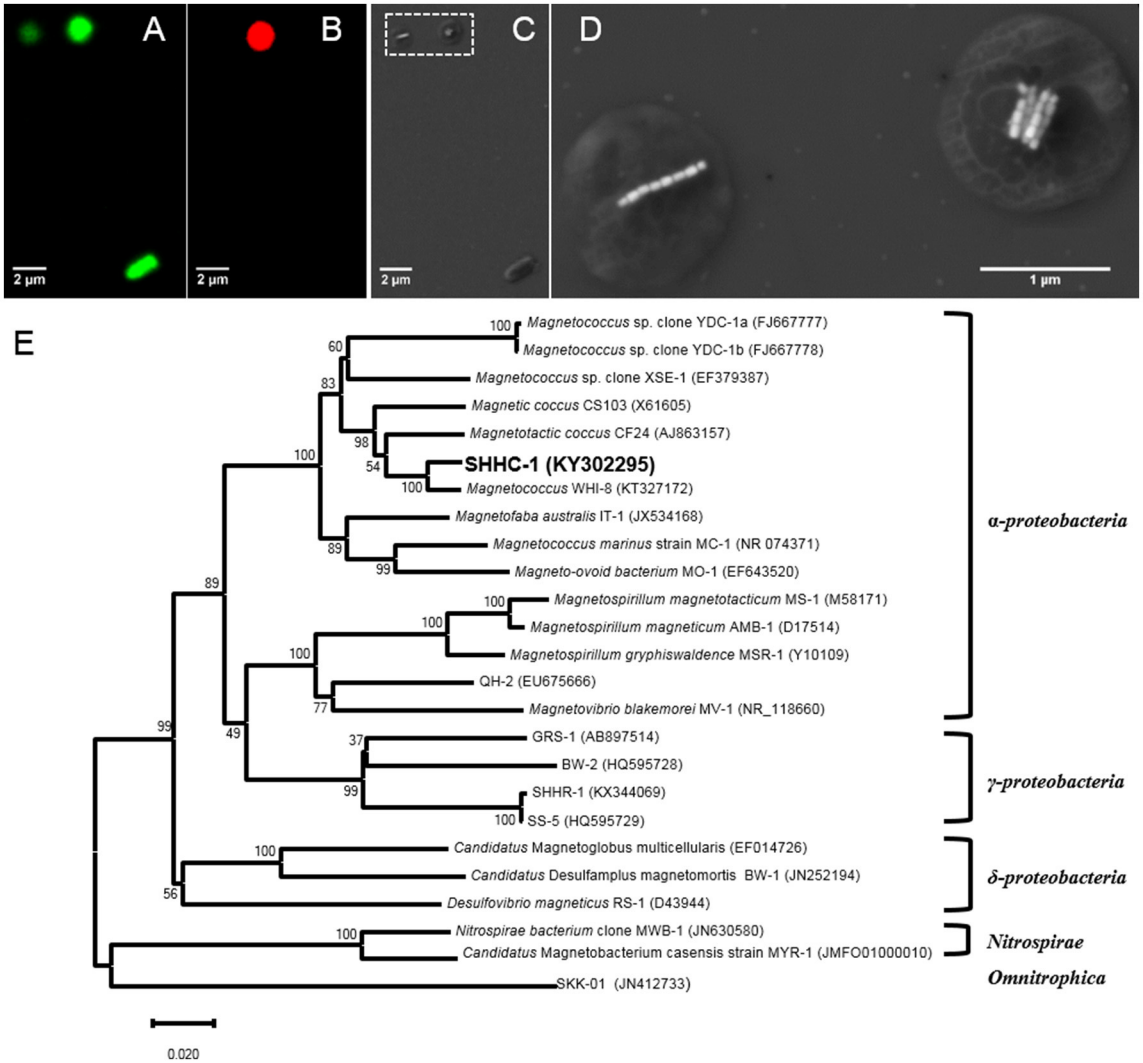
(A–D) Phylogenetic and structural identification of SHHC-1 cells by coupled FISH-SEM. **(A)** Fluorescence microscopy image of SHHC-1 cells *in situ* hybridized with the 5′-FAM-labeled universal bacterial probe EUB338. **(B)** Fluorescence microscopy image of SHHC-1 cells hybridized *in situ* with the 5′-Cy3-labeled SHHC-1-specific probe SHHC1228. **(C)** Low-magnification SEM image of the same field of view as in **(A)**. **(D)** High-magnification SEM images of two magnetococci cells outlined in image **(C)** by a white dashed square **(E)** Phylogenetic tree, based on 16S rRNA gene sequences, showing the position of strain SHHC-1 in the *Chromatiales* order of the *Alphaproteobacteria* class. Bootstrap values (higher than 50) at nodes are percentages of 1,000 replicates. Bar represents 2% sequence divergence.

To phylogenetically and structurally identify the strain SHHC-1, 16S rRNA genes of the magnetically enriched cells were amplified with a total of 30 clones being sequenced. Twenty-three 16S rRNA gene sequences with full-length had more than 99% of sequence identity. Coordinated FISH-SEM analysis using the SHHC1228 FISH probe designed to match the 23 identical 16S rRNA gene sequences was conducted on the magnetically enriched cells. As shown in Figures 3A–D, all bacterial cells, including the inner control *E. coli* cells, were positively labeled (green) by the 5′-FAM-labeled universal bacterial probe EUB338, while only the group of magnetococci with two bundle of magnetosome chains were positively labeled (red) by the 5′-Cy3-labeled SHHC-1-specific probe SHHC1228. This confirmed that the twenty-three 16S rRNA gene sequences corresponded to strain SHHC-1. Phylogenetic analyses based on the 16S rRNA gene shows that the strain of SHHC-1 affiliates with the *Alphaproteobacteria* class of the *Proteobacteria* phylum (Figure 3E). Moreover, it was phylogenetically close to (1) the uncultured magnetotactic coccus sp. clone WHI-8 (97% sequence identity), detected in intertidal sediments of the Xiaoshi Island in the North Yellow Sea (Weihai, China) (Chen et al., 2015), and (2) the uncultured magnetotactic coccus sp. CF24 (AJ863157) (95% sequence identity), detected in marine sediments in Germany (Flies et al., 2005). However, the bacterial and magnetosome morphologies of both WHI-8 and CF24 clones have not been described due to that both studies did not link their 16S rRNA gene sequences to the bacterial cells (Flies et al., 2005; Chen et al., 2015). Furthermore, to our knowledge, the morphological features and spatial arrangement of SHHC-1 magnetosomes have not been reported in any cultured and uncultured magnetococci. Therefore, SHHC-1 represents a novel magnetotactic coccus morphotype which is detected from the estuary of the Shihe River in Qinhuangdao City, eastern China.

### Crystal morphology of SHHC-1 magnetosomes

**Figure 4:**
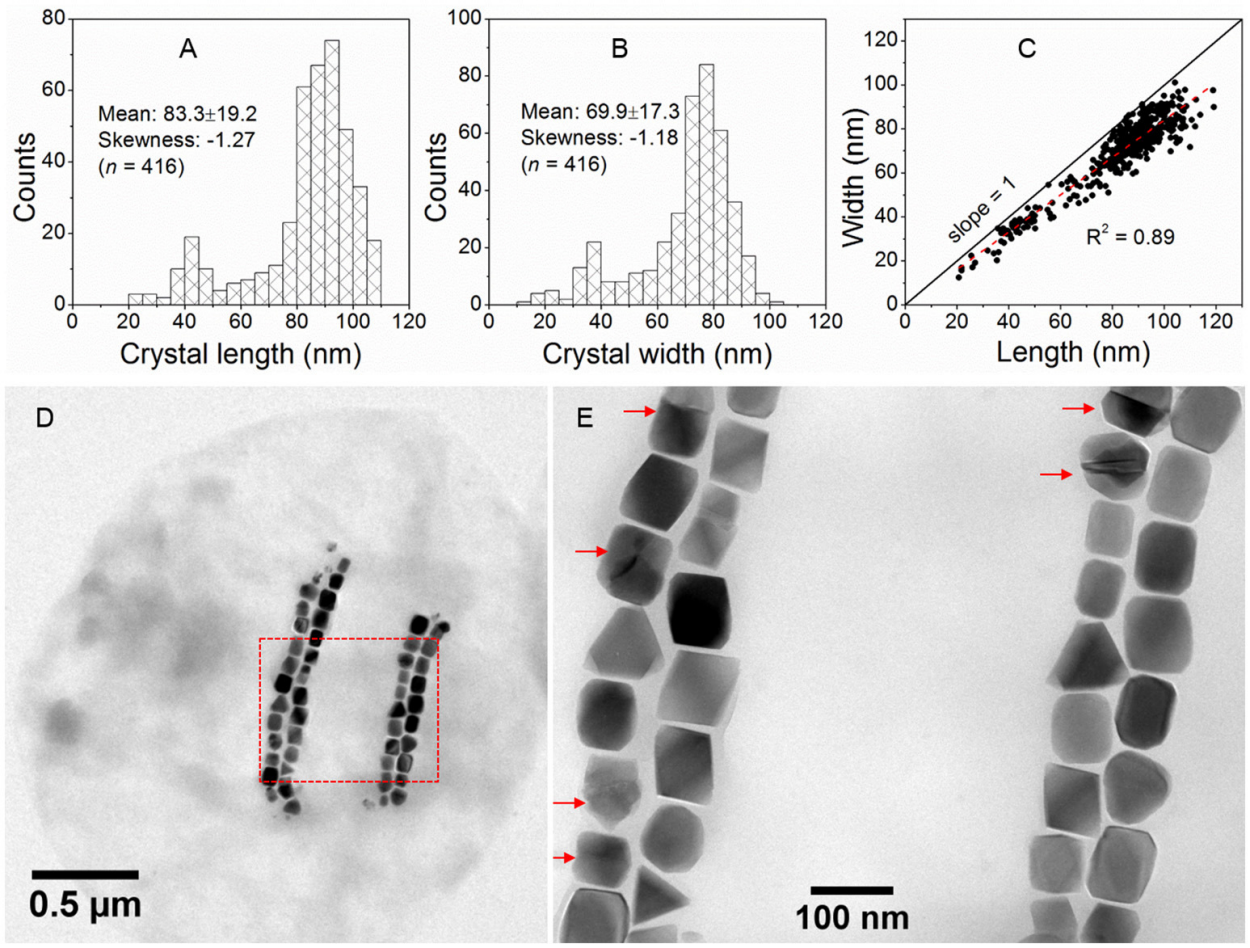
Morphological features of SHHC-1 magnetosomes. **(A,B)** Histograms of magnetosome length **(A)** and magnetosome width **(B)**. **(C)** Plot of crystal length vs. width of magnetosomes showing a linear relationship between crystal length and width of SHHC-1 magnetosomes. **(D)** Bright-field TEM image of one of the single SHHC-1 cells. **(E)** Close-up on the area outlined in image **(D)** by a red dashed rectangle. The red arrows indicate twin crystals of magnetosomes.

**Figure 5:**
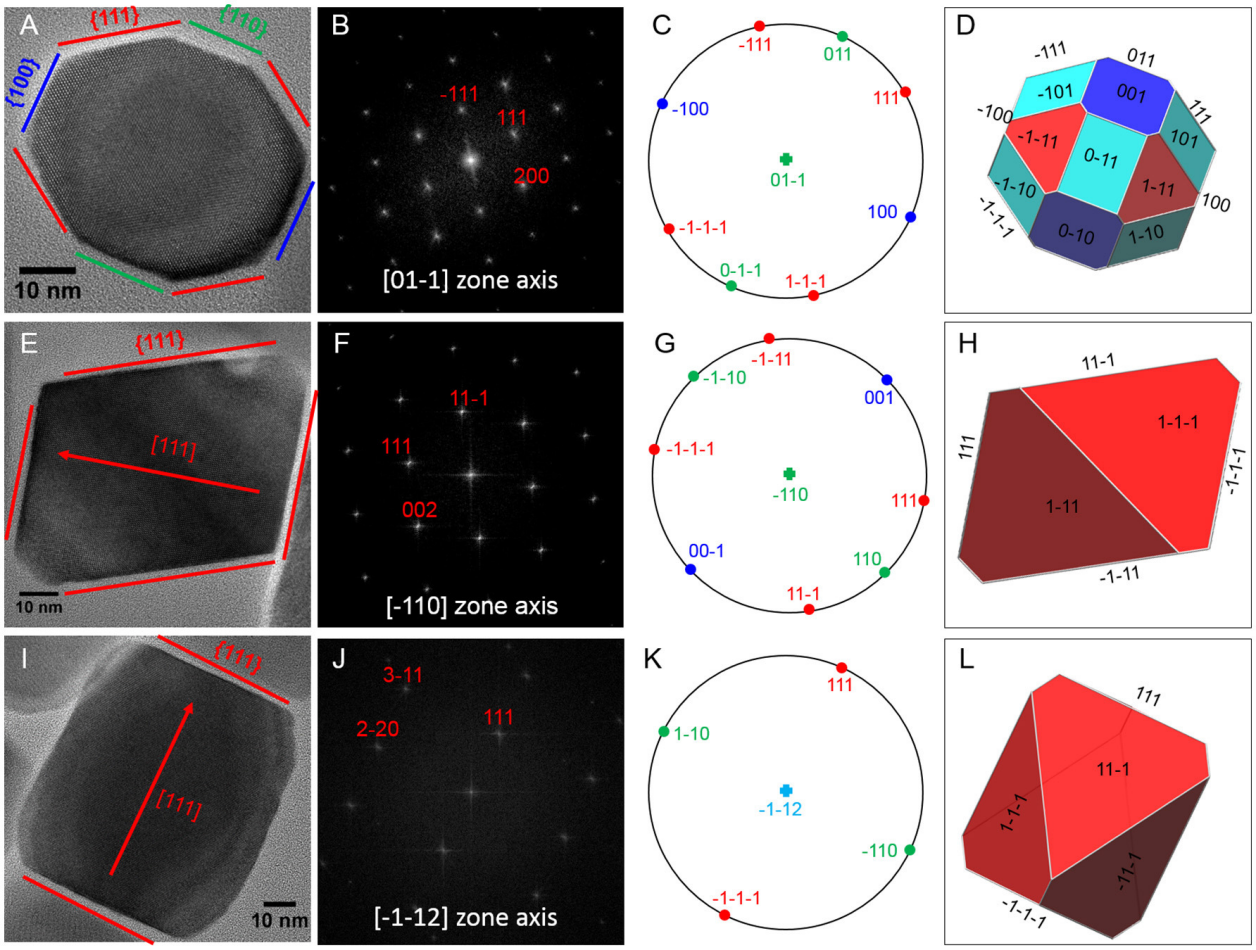
HRTEM analyses of three representative SHHC-1 magnetosomes: **(A)** an immature magnetosome recorded from a [0–11] zone axis, **(E)** a mature magnetosome recorded from a [–110] zone axis, and **(I)** a mature magnetosome recorded from a [–1–12] zone axis. The first column shows HRTEM images of individual magnetosomes **(A,E,I)**; the second column shows their corresponding indexed Fast Fourier Transforms (FFT) patterns **(B,F,J)**; the third columns shows stereographic projections **(C,G,K)**; and the forth columns shows the morphological modes **(D,H,L)**. For mature magnetosomes **(E,I)**, the outlines and lattice fringes of the magnetosomes in their HRTEM images are consistent with the octahedrons based only on {111} faces. The immature one **(A)** has a cuboctahedral-like shape based on {111}, {110}, and {100} faces.

**Figure 6:**
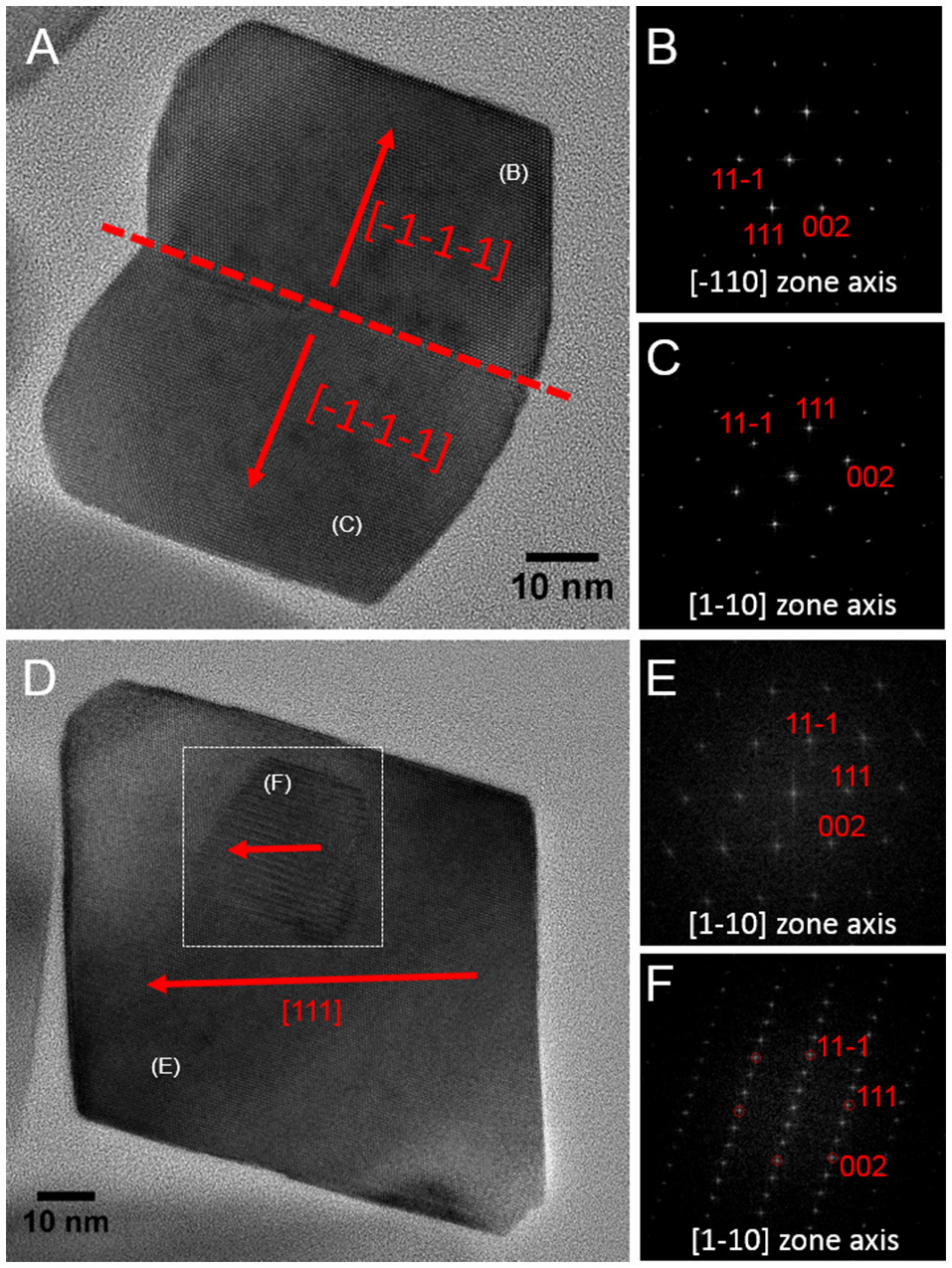
HRTEM images of defected crystals of SHHC-1 magnetosomes. **(A)** Bright Field HRTEM image of one twin magnetosome crystal with a straight contact (–1–1–1) plane **(A)**; corresponding indexed FFT patterns from the upper **(B)** and lower **(C)** parts of the crystal shown in **(A)**. **(D)** HRTEM of one defected magnetosome containing a stacking fault in it (indicated by white dashed square); corresponding indexed FFT patterns from the normal **(E)** and the defected **(F)** areas.

Magnetite crystals of SHHC-1 had an average length of 83.3 ± 19.2 nm and an average width of 69.9 ± 17.3 nm with a shape factor of 0.84 ± 0.07 (*n* = 416) (Figures 4A–C). Both the crystal length and width distributions were negatively skewed and the width/length ratio was nearly constant. These are typical features for prismatic and cuboctahedral magnetite magnetosomes (Devouard et al., 1998; Isambert et al., 2007). This indicates that magnetosomes within SHHC-1 grew homothetically within magnetosome membranes, a mechanism similar to that identified in known magnetotactic *Alphaproteobacteria* (Li et al., 2009; Jandacka et al., 2013). However, unlike what is generally observed in other cultured and uncultured magnetotactic *Alphaproteobacteria* where prismatic and cuboctahedral magnetosomes have a relatively consistent morphology (Pósfai et al., 2013a), there is a large diversity of magnetosome shapes in SHHC-1 even within the same cell (Figures 4D,E). Most particles had rectangular (~53%, *n* = 286) and rhomboidal (~26%) projections, whilst others displayed triangular (~6%) or irregular (~15%) shapes (Figure 4E). HRTEM observations performed on dozens of individual magnetosomes coupled with morphological modeling demonstrates that most particles with larger sizes (i.e., mature magnetosomes) are consistent with elongated octahedrons composed of only {111} faces (Figures 5E–L). Variations in the elongation ratios occur among individual magnetosomes (Supplementary HRTEM images). A few particles generally with smaller sizes (i.e., immature magnetosomes) have a cuboctahedral-like shape based on {111}, {110}, and {100} faces (Figures 5A–D). In addition, crystal defects including twining, multiply twining, and stack dislocations were very often observed within SHHC-1 magnetosomes (~15% of defect frequency, *n* = 286) (Figures 4E, 6).

## Discussion and conclusions

FISH with rRNA-targeted oligonucleotide probes has become a widely used method for the phylogenetic identification of MTB cells from various freshwater and marine sediments (Spring et al., 1992, 1995; Flies et al., 2005; Pan et al., 2008; Lin et al., 2011, 2012; Wang et al., 2013; Taoka et al., 2014). However, traditional optical and fluorescence microscopy cannot detect the structural features of magnetosomes due to their limitation in spatial resolution at around 0.2 μm. In the past decades TEM has made significant progress on both spatial and energy resolution, and now provides a powerful platform for obtaining simultaneous structural, compositional, and magnetic characterization of a single MTB cell down to the atomic scale (Pósfai et al., 2013b; Li and Pan, 2015; Li et al., 2015). On the other hand, TEM cannot provide phylogenetic affiliation information on the bacteria. This may explain why only a few uncultured MTB strains have been identified at the single-cell level both phylogenetically and structurally. In an early study, Spring et al. (1998) developed a method for linking the ultrastructure of enriched MTB cells with their 16S rRNA sequence *via* TEM of ultrathin sections that were hybridized *in situ* with digoxigenin- and fluorescein-labeled polynucleotide probes. The authors found one ovoid MTB from the Itaipu lagoon in Rio de Janeiro, that has Itaipu I 16S rRNA genotype and forms unusually large magnetosomes (Spring et al., 1998). Based on a targeted phylogenetic and ultrastructural analysis of micromanipulated single cells, Kolinko et al. (2012) detected a large ovoid cell, “SKK-01,” belonging to the proposed “*Candidatus* Omnitrophica” phylum. Here, the coupled FISH-SEM approach has successfully identified the SHHC-1 cells at the single-cell level from non-magnetotactic bacteria (i.e., *E. coli*) and from other magnetococci such as those forming single magnetosome chain (Figure 3). This demonstrates the feasibility and effectiveness of this coupled approach on those uncultured MTB cells that are dominant populations within the magnetically enriched cells, providing opportunities for the study of bacterial diversity and magnetosome biomineralization in uncultured magnetococci.

Magnetosomes formed by the cultured *M. marinus* strain MC-1 were reported to be elongated prismatic magnetites which can be idealized as hexagonal prisms elongated along a <111> direction and composed of six large {110} side faces, two large {111} end faces, and other small {110} and {100} truncated faces (Meldrum et al., 1993; Devouard et al., 1998). Within MC-1, magnetosomes were organized as a single chain with the chain direction parallel to the elongation direction of individual particles, i.e., [111] direction (Meldrum et al., 1993). In contrast, HRTEM observations performed on many individual magnetosomes coupled with morphological modeling demonstrates that most of mature magnetosomes produced by SHHC-1 are slightly elongated octahedrons only composed of {111} faces. This crystal habit is similar to that reported in strain IT-1 by Araujo et al. (2016). However, the spatial arrangement of magnetosomes within strain SHHC-1 are quite different from that in the cultured strains MC-1, MO-1, and IT-1. For instance, there are two bundle chains in SHHC-1 and one single chain in MC-1, MO-1, and IT-1 (Lefèvre et al., 2009; Bazylinski et al., 2013; Araujo et al., 2016; this study). Based on studies of cultured and uncultured MTB strains (Meldrum et al., 1993; Faivre et al., 2008; Lefèvre et al., 2011a; Li and Pan, 2012; Li et al., 2015), a correlation was proposed between the magnetosome mineral habits and the phylogenetic affiliations of MTB (Pósfai et al., 2013a). Here, the formation of octahedral magnetites and the arrangement of bundle magnetosome chains within SHHC-1, supports this correlation and indicates a species- or strain-specific mineral habits and spatial arrangement of magnetosomes.

Rather than MTB based biomineralization, nanocrystals of magnetite with octahedral, cubotachedral or prismatic shapes can also be produced by processes such as the co-precipitation of ferrous and ferric ions in aqueous solutions (Faivre et al., 2005), thermal decomposition of Mg–Fe carbonates (Golden et al., 2001), and extracellularly, biologically-induced mineralization by iron-reducing bacteria (Zhang et al., 1998; Vali et al., 2004). These non-MTB processes may produce magnetite crystals with some features (e.g., crystal habits, narrow size distribution in the single magnetic domain size range, pure chemistry) similar to those of magnetosomes. This makes the identification of magnetofossils from ancient sediments or rocks more complicated and sometimes misleading (Golden et al., 2004; Benzerara and Menguy, 2009; Jimenez-Lopez et al., 2010). In addition, the crystal shape, grain size and chemical composition of magnetosomes can be modified by growing MTB cells under high oxygen environments or within the culture medium rich in some other transition metals or inorganic chemicals (Devouard et al., 1998; Taylor and Barry, 2004; Faivre et al., 2008; Lefèvre et al., 2011b; Li and Pan, 2012; Li et al., 2016). Moreover, genetic factors may also have an impact on the formation of irregular shaped magnetite magnetosomes (Lohsse et al., 2014). Our study shows that some uncultured MTB produce magnetosome magnetites with octahedral and irregular shapes and with a high frequency of twin crystals. On the one hand, these features do not meet some of the criteria of biogenicity enacted for magnetofossils, such as crystallographic perfection and unusual crystal morphology (Thomas-Keprta et al., 2000). This indicates that besides crystal morphology, other robust criteria such as their chain structure, chemical composition, and the co-presence of reliable magnetofossils (e.g., bullet-shaped SD magnetites) should be carefully tested in order to precisely identify magnetofossils (Li et al., 2013). On the other hand, the observed variety of morphologies and the high frequency of observed crystal defects indicate that the magnetosome formation within the strain SHHC-1 might be less biologically controlled or that they were affected by unfavorable environments in which they live in. Therefore, more coupled fluorescence and electron microscopy studies on natural MTB cells, as well as systematic studies on magnetosome biomineralization within cultured and uncultured MTB are needed to better understand the correlation between magnetosome mineral habits and MTB phylogenies and/or habitats. This has important implications on the magnetosome biomineralization and further understanding magneto-aerotaxis processes.

## Nucleotide sequence accession numbers

The sequence obtained in this study was deposited in GenBank under accession number KY302295.

## Author contributions

JL, ZC, and YP designed the research. HZ, FW, PL, WL, and CW prepared samples and carried out microbiological experiments. JL, NM, KB, and EL carried out TEM experiments. JL, HZ, and FW performed FISH-SEM experiments. JL and HZ prepared the manuscript.

### Conflict of interest statement

The authors declare that the research was conducted in the absence of any commercial or financial relationships that could be construed as a potential conflict of interest.
