# Supplementary material for: Magnetotactic Coccus Strain SHHC-1 Affiliated to Alphaproteobacteria Forms Octahedral Magnetite Magnetosomes
Source: Front Microbiol. 2017 May 30;8:969. doi: 10.3389/fmicb.2017.00969 (PMC5447723; doi:10.3389/fmicb.2017.00969)
Supplement: Supplementary file 2 [file Presentation1.PDF]

Supplementary Information

----HRTEM images and morphology modelling of SHHC-1 magnetosomes

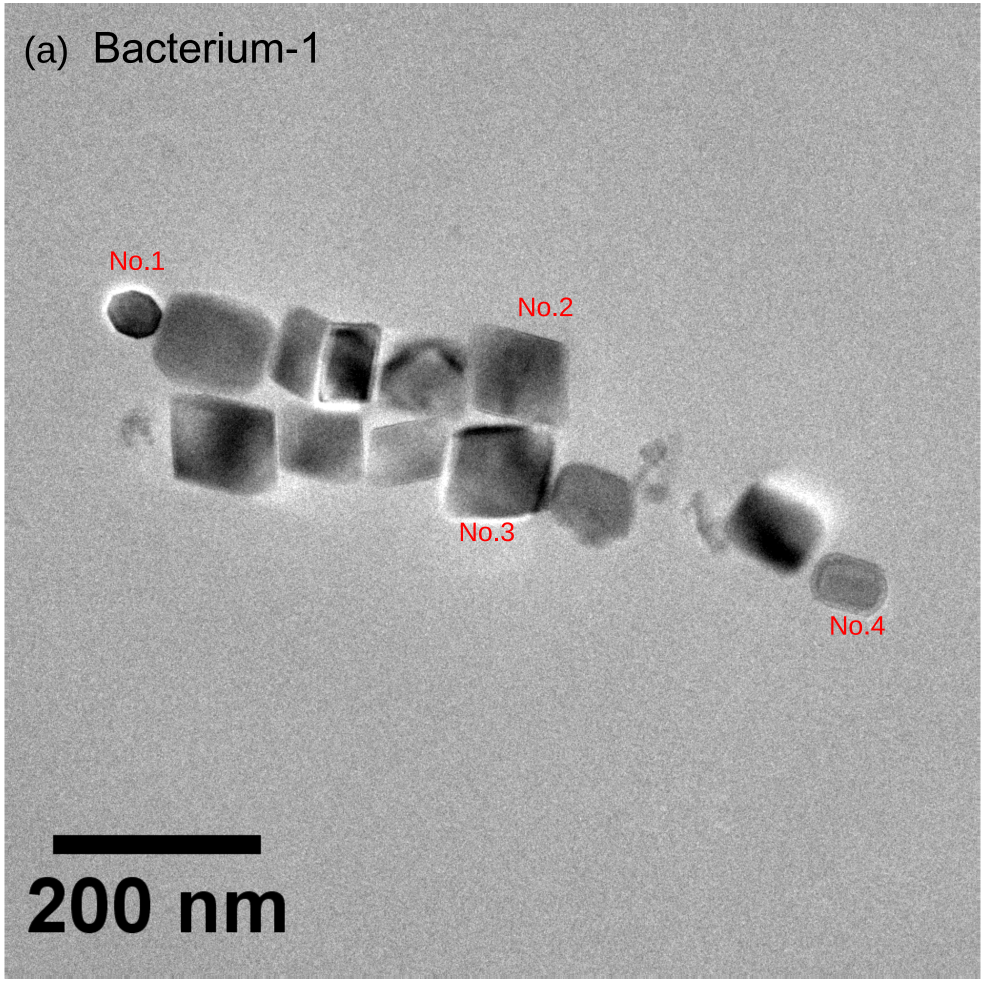

<table>
<tr><th>Particle No.</th><th>HRTEM image</th><th>FFT pattern</th><th>Stereographic projection</th><th>Morphology model</th></tr>
<tr><td>No.1</td><td>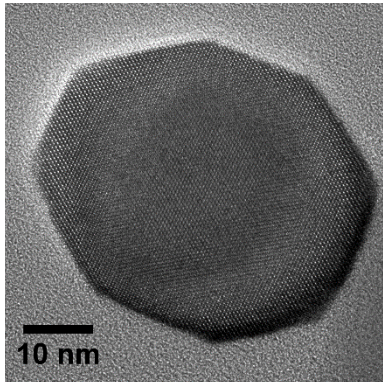
</td><td>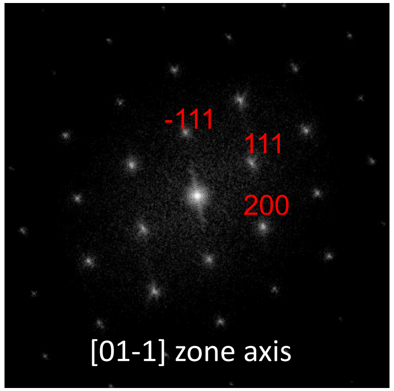
</td><td>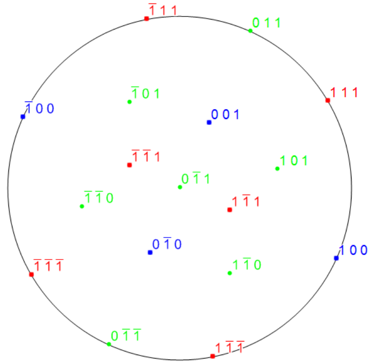</td><td>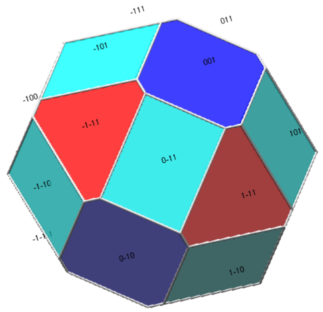
{111}+{110}+{100}</td></tr>
<tr><td>No.2</td><td>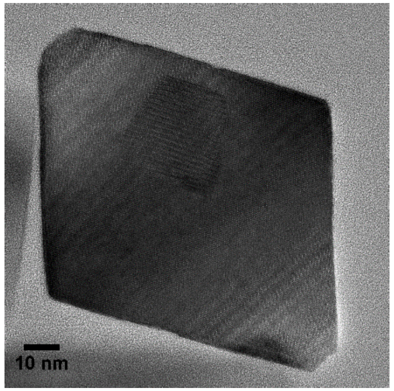
</td><td>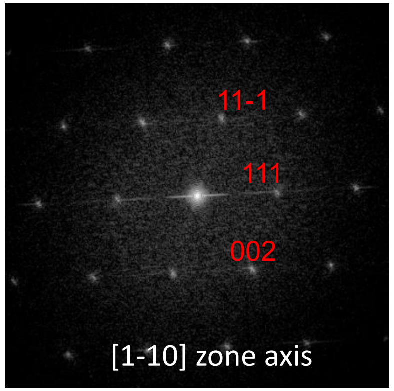
</td><td>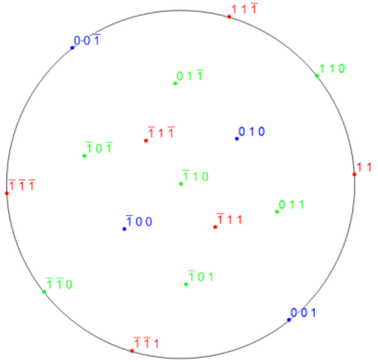</td><td>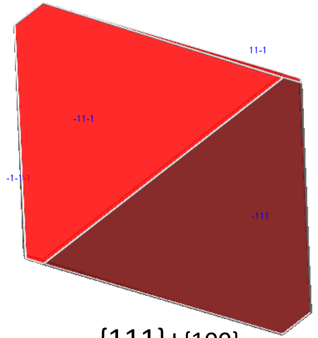
{111}+{100}</td></tr>
<tr><td>No.3</td><td>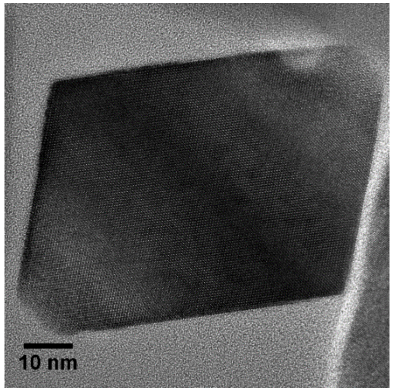
</td><td>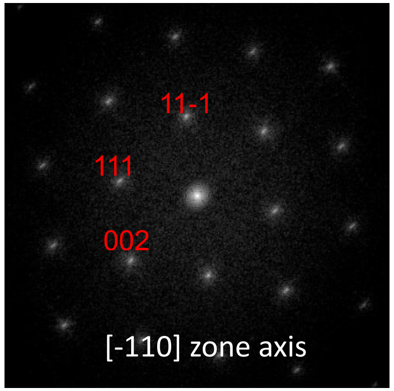
</td><td>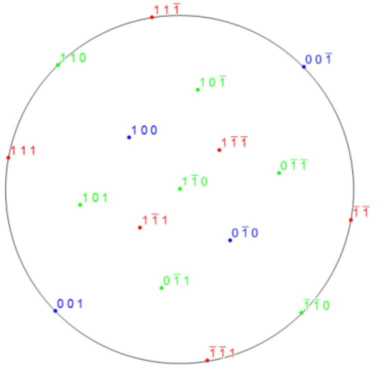</td><td>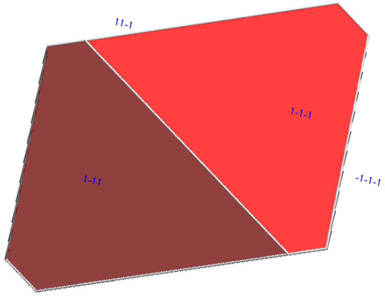</td></tr>
</table>

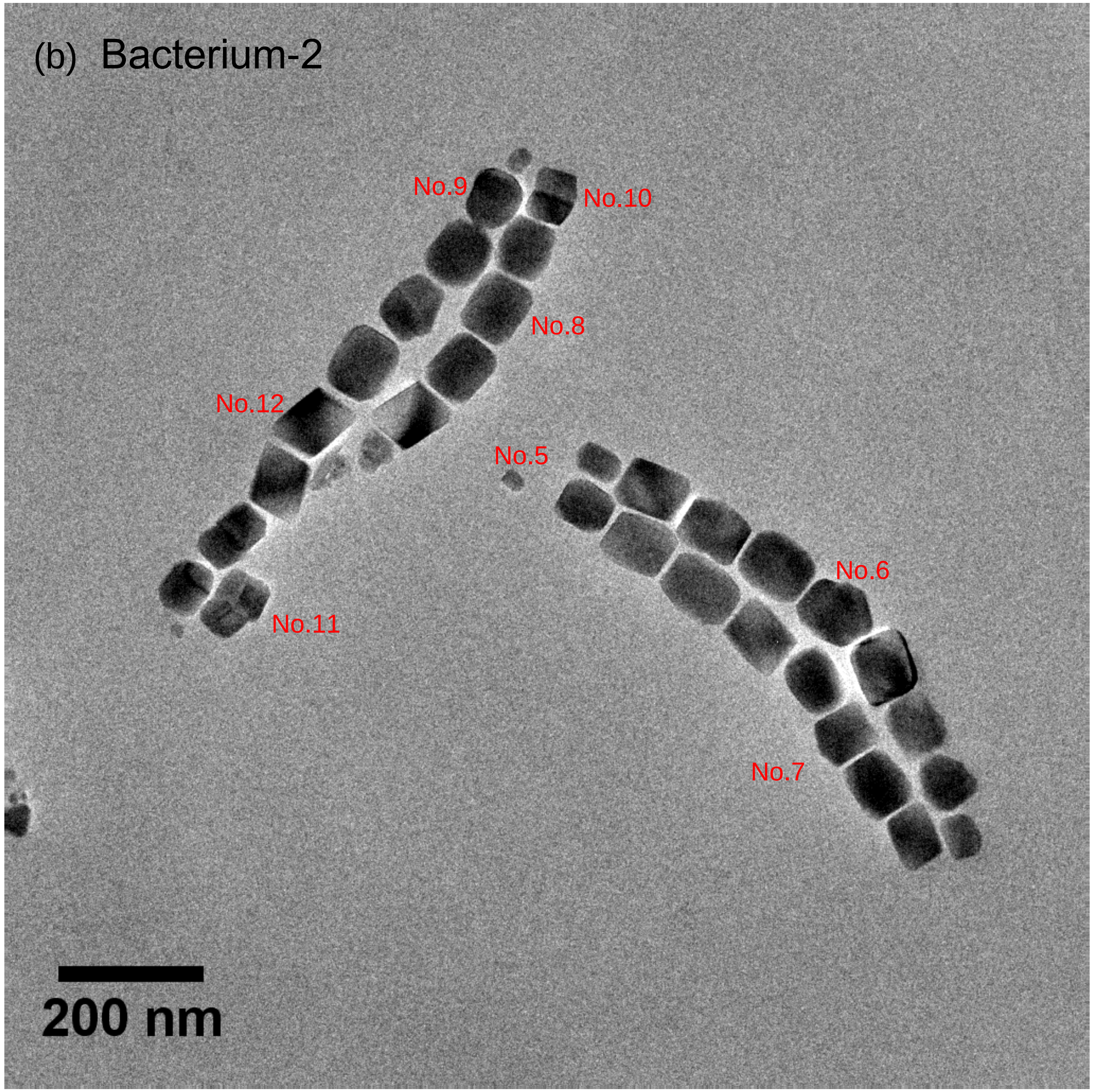

(b) Bacterium-2
No.9
No.10
No.8
No.12
No.5
No.6
No.11
No.7
200 nm

Particle No.	HRTEM image	FFT pattern	Stereographic projection	Morphology model
No.5	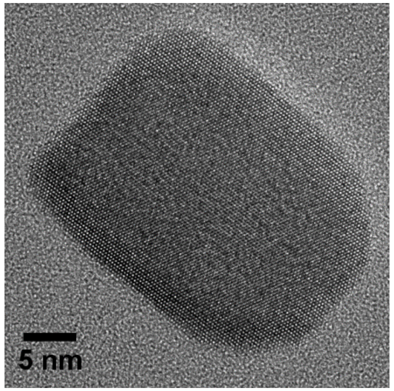	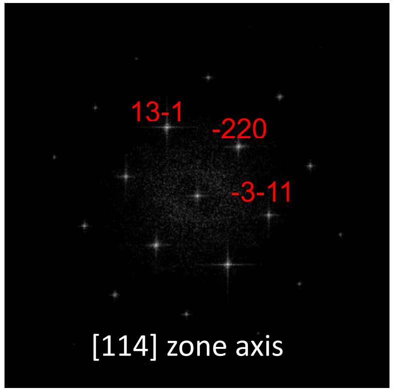	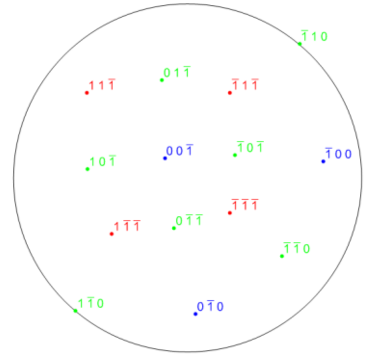	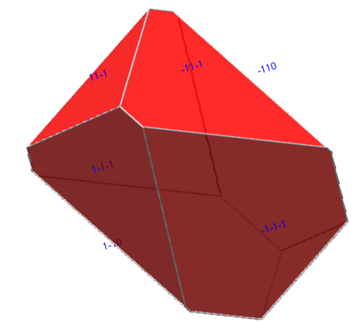
No.7	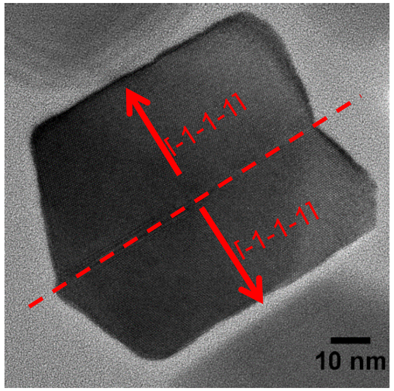	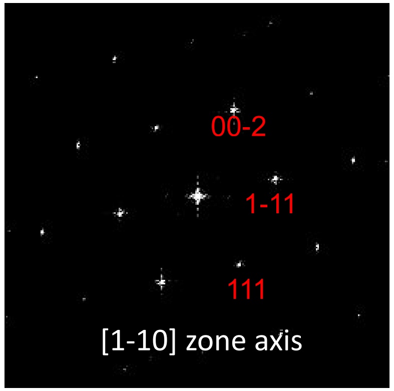	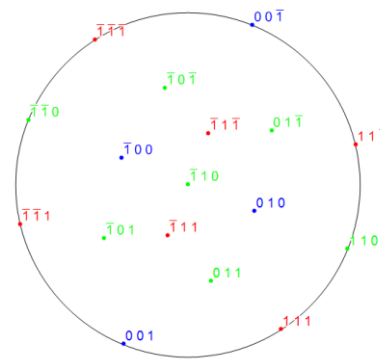	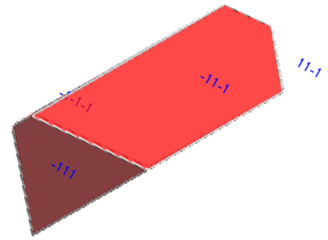
		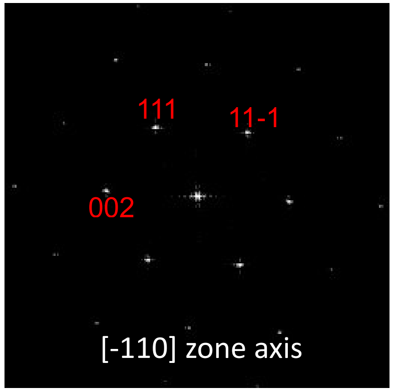	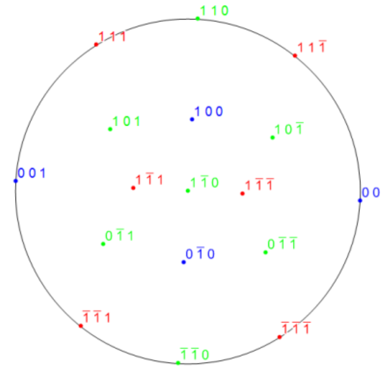	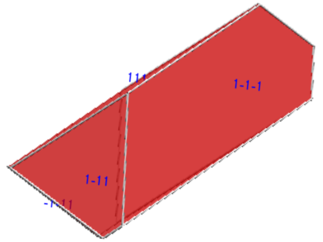

No.8	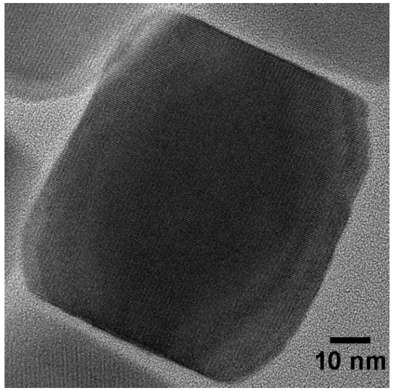 10 nm	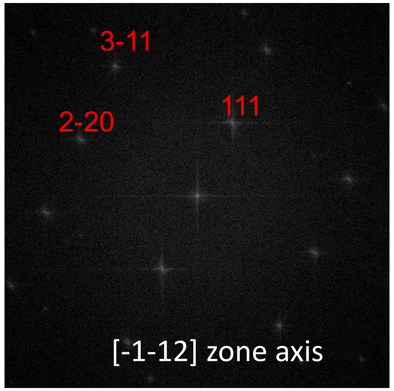 3-11<br>2-20<br>111<br>[-1-12] zone axis	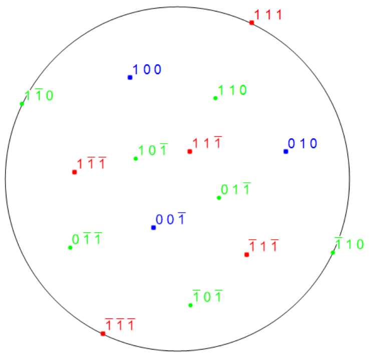	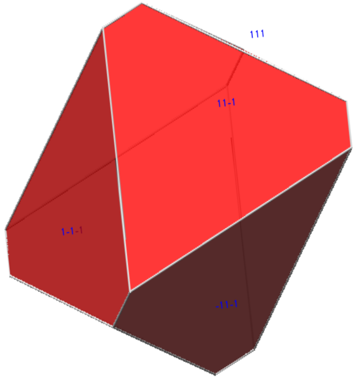
	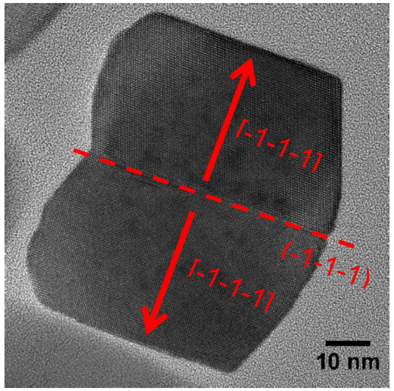 [-1-1-1]<br>(-1-1-1)<br>[-1-1-1]<br>10 nm	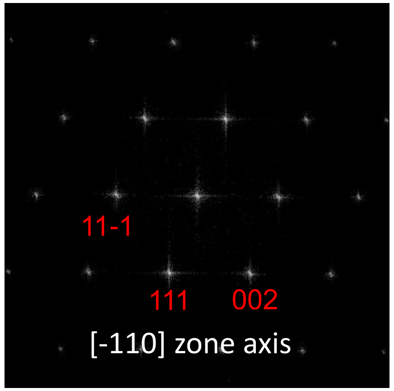 11-1<br>111<br>002<br>[-110] zone axis	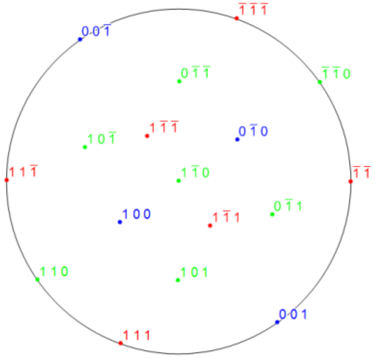	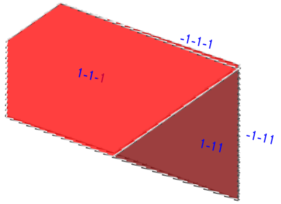
		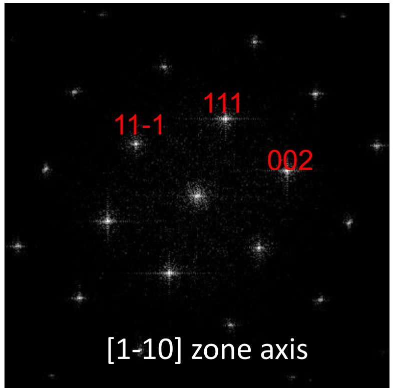 111<br>11-1<br>002<br>[1-10] zone axis	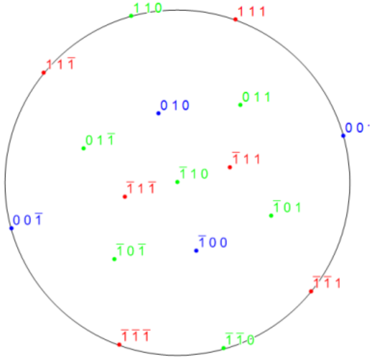	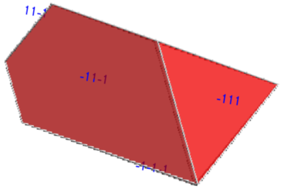

<table>
<tr>
<td></td>
<td>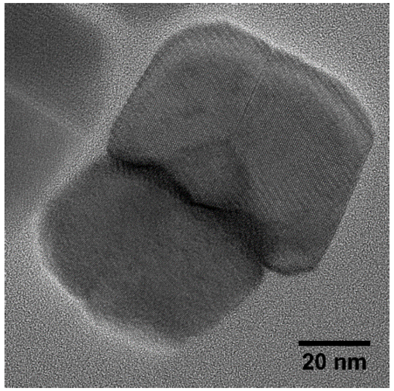</td>
<td>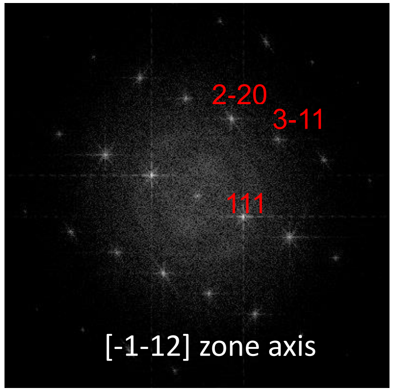</td>
<td>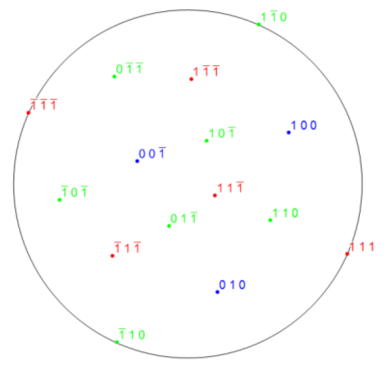</td>
<td>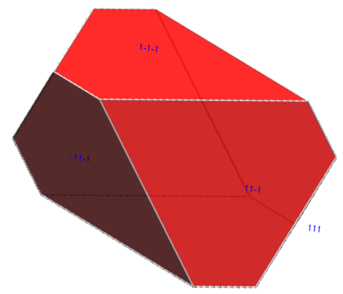</td>
</tr>
<tr>
<td>No12</td>
<td>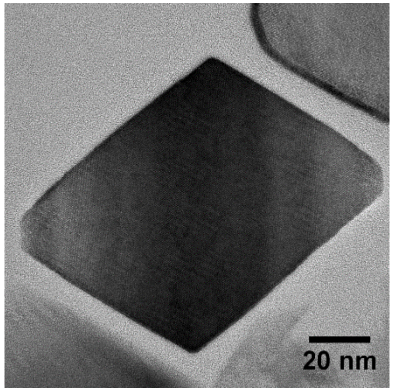</td>
<td>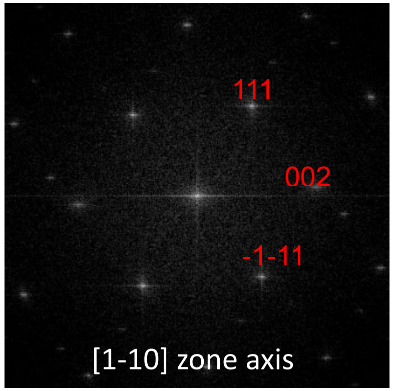</td>
<td>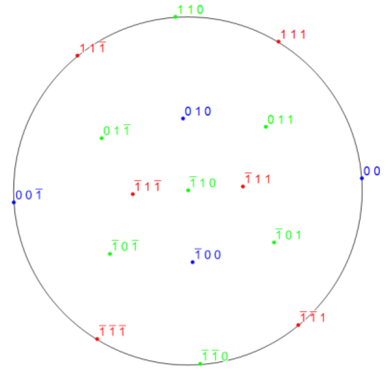</td>
<td>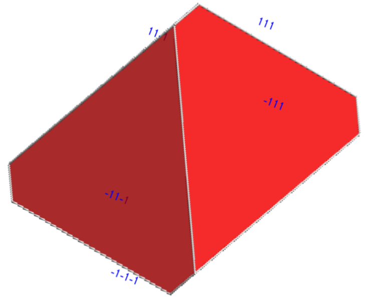</td>
</tr>
</table>
